# Endometriosis and Uterine Fibroids (Leiomyomata): Comorbidity, Risks and Implications

**DOI:** 10.3389/frph.2021.750018

**Published:** 2021-10-26

**Authors:** Outi Uimari, Hannah Nazri, Thomas Tapmeier

**Affiliations:** ^1^Department of Obstetrics and Gynecology, Oulu University, Oulu, Finland; ^2^PEDEGO Research Unit (Research Unit for Pediatrics, Pediatric Neurology, Pediatric Surgery, Child Psychiatry, Dermatology, Clinical Genetics, Obstetrics and Gynecology, Otorhinolaryngology and Ophthalmology) and Medical Research Center Oulu, Oulu University Hospital, Oulu, Finland; ^3^Endometriosis CaRe Centre, Nuffield Department of Women's and Reproductive Health, University of Oxford, Oxford, United Kingdom; ^4^Department of Obstetrics and Gynaecology, Monash University, Clayton, VIC, Australia

**Keywords:** endometriosis, uterine fibroids, leiomyoma, comorbidity, symptoms

## Abstract

Uterine Fibroids (leiomyomata) and endometriosis affect millions of women world-wide. Although aetiology and natural history of the conditions are markedly different, symptoms can overlap and make differential diagnoses necessary, often using invasive methods such as laparoscopy. Considerable comorbidity exists between the two conditions and needs to be taken into account when treating fibroids and/or endometriosis. The genetic foundations of both uterine fibroids and endometriosis remain to be fully understood but recent evidence suggest common underpinnings. Here, we discuss the comorbidity of uterine fibroids and endometriosis and the implications for diagnosis, treatment and risks.

## Introduction

The uterus is indispensable for the growth of embryos to term. Even as efforts to replace it by technology are under way (1), it is arguably the organ least replaceable in human reproduction. World-wide, between 48.5 million (2) and 72.4 million (3) couples of reproductive age suffer from infertility, with uterine conditions common causes. Apart from infertility, symptoms of uterine disease are abdominal pain, pain during sex (dyspareunia), and heavy menstrual bleeding (HMB). Most prominent amongst the conditions evoking these symptoms are uterine fibroids (leiomyomata) and endometriosis, and their comorbidity has been addressed by various large-scale observational studies in the last decade. At the same time, increasing access to genetic information has enabled researchers to start unravelling the genetic underpinnings of both conditions.

Here, we review the evidence for comorbidity between both endometriosis and uterine fibroids, and the associated risks and implications.

## Symptoms

**Figure 1:**
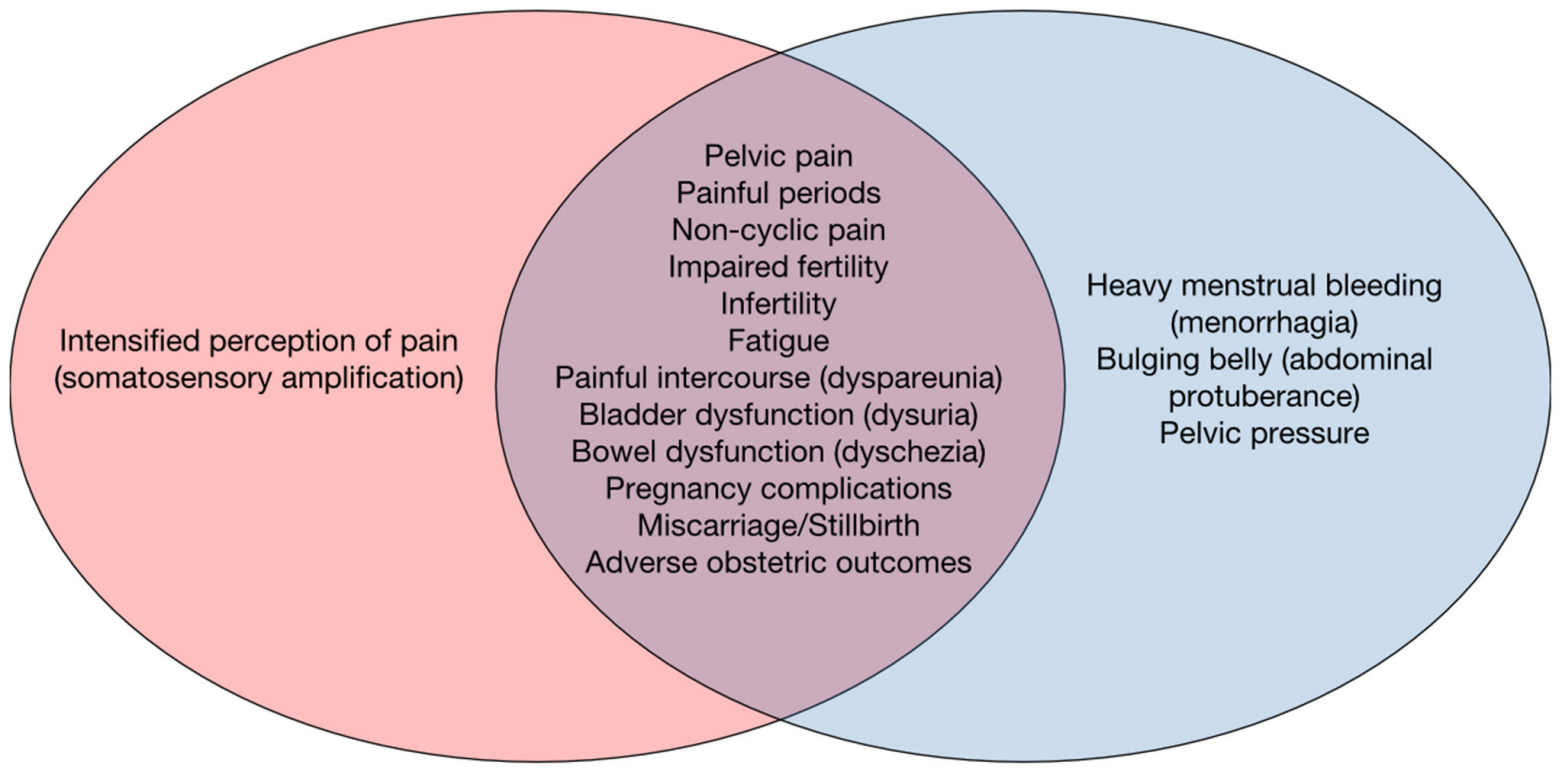
The symptoms of uterine conditions, endometriosis and uterine fibroids overlap. The non-specificity of abdominal and uterine symptoms makes it challenging to differentially diagnose endometriosis (left, red) and uterine fibroids (blue, right) without imaging or even surgical means.

Symptoms arising from uterine disease are manifold and overlapping between endometriosis (4) and uterine fibroids (5) (Figure 1): Both conditions—and we include adenomyosis as a subtype of endometriosis growing within the uterus—can manifest in severe pelvic pain, painful periods and non-cyclic pain, impaired fertility or outright infertility, fatigue, painful intercourse (dyspareunia), and bladder and bowel dysfunction (dysuria and dyschezia, respectively). Uterine fibroids in addition provoke heavy menstrual bleeding (HMB) as a prominent symptom in ~30% of patients (6, 7) and can further lead to feelings of pelvic pressure and bulging abdominal protuberances (8). Endometriosis on the other hand canlead to an enhanced somatosensory perception accompanied by changes in central nervous pain processing, further sensitising patients to the pain symptoms of the condition (9, 10). Additionally, several other conditions need to be considered in differential diagnostics such as diseases of the bowel and the urinary tract, or musculoskeletal conditions (11). This makes differential diagnosis challenging, and together with poor awareness of endometriosis, both within the general public and by healthcare professionals, leads to long delays with regards to the diagnosis of endometriosis (12). While endometriosis generally requires laparoscopy to confirm the diagnosis, uterine fibroids are readily seen in ultrasound (US) imaging (5).

## Diagnosis

The onset of endometriosis is in adolescence or early adulthood (13). A clinical diagnosis of endometriosis can be indicated by moderate to severe dysmenorrhea that causes absenteeism from school or work (14). A positive family history on first degree relatives affirms the suspicion (15). Typical findings on clinical examination are tenderness or drastic pain on the vaginal posterior fornix caused by peritoneal lesions on the Pouch of Douglas or rectovaginal lesions. Large endometriomas can be detected by bimanual palpation. Endometriomas in general however are diagnosed by transvaginal ultrasound scan (TV-USS). Rectovaginal lesions can in principle also be detected by TV-USS (16), however, other lesions, e.g., on the bowel, may be diagnosed by MRI. The definitive diagnosis of endometriosis however requires surgery, typically laparoscopy or laparotomy, but indications for operative treatment need to be carefully assessed, and the expected outcome (an increased quality of life and/or fertility) should outweigh the risks [injury to bowel, bladder, ureters, loss of ovarian reserve, adhesions, haemorrhagia, and post-operative infection (17)].

In contrast to endometriosis, uterine fibroids are typically diagnosed in women of at least 40 years of age (7). Fibroids present in single or multiple numbers with size ranging from millimetres to up to 20 cm in diameter (18). An enlarged uterus or a uterine-related tumour resistance can be detected by bimanual palpation. Again, TV-USS brings accuracy to further investigating fibroids, such as their morphology, size, number and location within the uterus. Fibroids that distort the uterine cavity can be detected by hysterosonography. Large or multiple fibroids impair the ultrasound view by leaving shadows and thus fading the tissue borders. MRI can be an alternative method to gain more information on uterine tumours. The definitive diagnosis is only reached after pathological examination and analysis of the tissue. Fibroids are the most common indication for hysterectomy (5). However, they are seldom the sole cause of infertility (19).

## Incidence and Comorbidity

Endometriosis affects ~10% of women of reproductive age, up to 170 million world-wide (4). However, due to the difficulty of diagnosing, lack of awareness and the absence of clinically useful biomarkers, the real incidence is likely substantially higher (20). Uterine fibroids show an incidence of ~70% in women aged 35–49 years old, with a higher incidence of 80% found in black women [United States (21)]. In the United States, fibroids are cited to be the cause for over 50% of hysterectomies (22), and direct costs for their treatment is estimated between 4 and 9 billion USD (23).

With prime symptoms like pelvic pain and infertility overlapping between endometriosis and uterine fibroids, it is important to investigate the degree of comorbidity between the two, as treatment of one without the other, if present, could fail to eliminate the pelvic pain or infertility symptoms and make further treatment or even surgeries necessary.

To review the degree of comorbidity between the conditions, we conducted a literature search on PubMed using the terms “uterine fibroids,” “endometriosis” and “comorbidity” in conjunction, and found several studies that have addressed comorbidity between endometriosis and uterine fibroids in the recent decades (Table 1). The first studies used retrospective analyses of clinical data at local medical centres to report statistics on comorbidity (19, 24–27); these were followed by larger-scale retrospective studies of medical insurance claims data (29, 30). Finally, the results from long-term prospective cohort studies are emerging (28, 31), which offer the exciting possibility of monitoring disease incidence, development, and comorbidity over the years.

**Table 1:** Comorbidity of endometriosis and uterine fibroids.

**Study**	***N***	**Comorbidity**	**References**
Italian Group for the Study of Endometriosis (retrospective)	3,684 overall, 1,880, with fibroids diagnosed	12% (95% CI 10–14) of fibroids comorbid with endometriosis	(24)
Stanford/Atlanta Study (retrospective)	131, with fibroids diagnosed	86% had endometriosis; 4.6% had adenomyosis; 9.2% had fibroids without comorbidity.	(25)
Lublin Study (retrospective)	233, adenomyosis diagnosed	57.9% comorbid with uterine fibroids.	(26)
Oulu Study (retrospective)	558 overall, 182, endometriosis diagnosed, 240, with fibroids diagnosed, 183, control group (no diagnosis of endometriosis or fibroids)	25.8% endometriosis comorbid with fibroids (9.3% in controls); 19.6% fibroids with endometriosis (5.5% in controls)	(27)
Japan Nurses' Health Study (prospective)	49 927 overall	OR for comorbid endometriosis and fibroids 4.47 (95% CI 4.09–4.87)	(28)
Palo Alto Study (retrospective)	• 244 overall • 208, with fibroids diagnosed	• 87.1% fibroids comorbid with endometriosis • 4.3% fibroids comorbid with adenomyosis	(19)
Korean Health Insurance Review and Assessment (HIRA) Study (retrospective)	61,516 overall	• OR for comorbid endometriosis and fibroids 0.2 (*P* = 1.17E-15) • OR for comorbid adenomyosis and fibroids 0.3 (*P* = 7.85E-12)	(29)
Minnesota Study (insurance claims, retrospective)	26,961 endometriosis cases/107,844 matched controls	• aHR for comorbid endometriosis and fibroids 3.9 (95% CI 3.6–4.1, *P* < 0.001) • aHR for laparoscopically confirmed endometriosis and fibroids 4.0 (95% CI 3.5–4.7, *P* < 0.001)	(30)
Taiwan NHIRD Study (prospective)	31,239 fibroid cases/124,956 matched controls	aHR for comorbidity with endometriosis 6.44 (95% CI, 6.18, 6.72)	(31)

*CI, confidence interval; OR, odds ratio; aHR, adjusted hazard ratio*.

The degree of comorbidity found in the retrospective studies varies but is substantial: The first oft-cited study was conducted in Italy in 1994 and reported the presence of endometriosis in 12% (95% CI 10–14%) of patients with confirmed uterine fibroids (24). In contrast, a study conducted in 2010 at a medical centre in the US reported much higher incidence of comorbidity, with 86% of confirmed cases of uterine fibroids having comorbidity with endometriosis; however, the sample size was considerably smaller (25). A study from Lublin in Poland in the same year reported 57.9% of comorbidity with uterine fibroids in adenomyosis patients, again confirming that the symptoms patients experience are often not solely due to a single cause (26). Ten years ago, a retrospective study comparing endometriosis patients and uterine fibroid patients to controls was conducted at a medical centre in Oulu, Finland, and discovered that 25.8% of endometriosis patients were comorbid with uterine fibroids, in comparison to 9.3% of endometriosis-free controls (2.8-fold incidence), while 19.6% of fibroid carriers were comorbid with endometriosis, in comparison to 5.5% in controls (3.6-fold incidence) (27). Since then, prospective studies have started reporting results, such as the Japan Nurses' Health Study (28), which reported an odds ratio of 4.47 (95% CI 4.09–4.87) for comorbidity of endometriosis with uterine fibroids.

Another retrospective study at a US centre in 2016 reported a similarly high comorbidity of 87.1% in uterine fibroids patients with endometriosis (19) as the earlier 2010 US study (25), while also reporting an additional 4.3% of comorbidity in uterine fibroid patients with adenomyosis.

Comorbidity incidence is much higher in retrospective studies conducted in medical referral centres than is reported from medical insurance data. Several studies have retrospectively analysed data on insurance claims, and an interesting finding was reported from Korean data in a 2017 study, which surprisingly reported a decrease in risk of developing uterine fibroids in both endometriosis (OR 0.2, *P* = 1.17E-15) and adenomyosis patients (OR 0.3, *P* = 7.85E-12) (29).

In contrast, a Minnesota study of insurance claim data conducted in 2018 found an increased risk of endometriosis comorbidity with uterine fibroids in laparoscopically confirmed cases of endometriosis (aHR 4.0, 95% CI 3.5–4.7, *P* < 0.001). Prospective studies have the advantage of investigating the presence of endometriosis or uterine fibroids at the time of surgery for either of these conditions, as in a very recent 2021 study of Taiwanese women, with 14 years of follow-up (31). This study confirmed that the presence of uterine fibroids increases the risk of developing endometriosis and reported an adjusted hazard rate of 6.44 (95% CI 6.18–6.72).

Patients with comorbidity of fibroids and endometriosis tend to have more severe pelvic pain than those without endometriosis, with a similar effect on fertility (19). The retrospective design and the specifics of the health systems of many of these studies could introduce a population bias, also the location in referral centres, which are frequented predominantly by patients suffering from uterine disease and pelvic pain. The insurance claim studies largely avoid these limitations but might lack clinical diagnosis of cases.

Since endometriosis and uterine fibroids develop along different trajectories and temporal incidence, with endometriosis appearing earlier in life than fibroids, endometriosis could already have been established before it was discovered on the occasion of fibroid-related diagnostic or surgical interventions. Similarly, the distribution of stage I/II endometriosis vs. stage III/IV might be different in uterine fibroids patients compared to the general population as patients with mild disease often undergo treatment by medication first before seeking surgical means, which would allow both endometriosis and fibroids to progress.

Observational studies like these cannot determine whether endometriosis and uterine fibroids arose through a common mechanism, or whether one subsequently led to the development of the other. However, given the substantial comorbidity between the conditions, it might be beneficial to factor in surgery for one condition when addressing the other, so as to avoid the need for repeating surgical procedures. To unravel causal mechanisms, genetic studies are needed, ideally in combination with prospective collection of cohort data.

## Genetic Underpinnings

Apart from the same genes being constitutively active or inactive by mutation, similarly activated pathways could also lead to an increase in morbidity risk for one condition if the patient already presents with the other one. Consequently, new studies try to account for the importance of a combined patient cohort and recruit participants with either disease into a comprehensive downstream analysis of genotypes, to be correlated with clinical phenotypes (32).

Interestingly, one study found that patients with smaller fibroids were more likely to have severe endometriosis than those with large fibroids (25). Given the difference in cellular composition of small vs. large fibroids (33) and different genetic conditions likely underlying their development (34), it is conceivable that these differences would also be mirrored in the manifestation of endometriosis severity.

Recent evidence from classic and genetic epidemiology points to an association of both conditions (35, 36). The genetic drivers of uterine fibroids have been elucidated in the past two decades as mutated MED12, HMGA2, FH deficiency and Col4A5/A6 mutations (5). However, changes in chromatin accessibility due to deficient histone deposition have now also been described as an enabling mechanism (37).

For endometriosis, the underlying genetic changes are much less clear: Owing to the complex nature and multifaceted aspect of endometriosis, long lists of risk factor genes carrying germline mutations that potentially contribute to endometriosis are identified, with the “hits” followed up experimentally (38). The overlap between the downstream targets of the mutations and pathomechanisms supports the notion of a combined risk of comorbidity that springs from underlying genetic factors, and two recent meta-analyses of genome-wide association studies (35, 36) describe four risk factor alleles previously associated with endometriosis as risk factors for uterine fibroids, too (Table 2).

**Table 2:** Risk alleles for the development of uterine fibroids previously associated with endometriosis.

**Gene ID**	**References**	**Locus/Position**	**Lead SNP**	**P_meta_**	**OR (95% CI)**
*WNT4, CDC42*	(35)	chr1:22096228	rs10917151	5.1 × 10^−14^	1.12 (1.09–1.16)
	(36)	1p36.12	rs7412010	2.4 × 10^−29^	1.13 (1.11–1.16)
*GREB1*	(35)	chr2:11524625	rs148143917	8.1 × 10^−10^	0.74 (0.67–0.82)
	(35)	chr2:11562535	rs10929757	8.1 × 10^−12^	0.92 (0.90–0.94)
	(36)	2p25.1	rs35417544	2.3 × 10^−19^	1.09 (1.07–1.10)
*SYNE1, ESR1*	(35)	chr6:152241136	rs58415480	9.0 × 10^−24^	1.18 (1.14–1.22)
	(36)	6q25.2	rs58415480	1.9 × 10^−54^	1.19 (1.17–1.22)
*FSHB*	(35)				
	(36)	11p14.1	rs11031006	5.7 × 10^−15^	1.10 (1.07-1.12)

*SNP, single nucleotide polymorphism; OR, odds ratio, position according to hg38*.

Both studies utilised the UK biobank (UKBB) as one of the largest repositories of genetic material in combination with epidemiological data but combined their analyses with different datasets in addition. However, both studies describe variants in loci on chromosome 1 encoding for *CDC42* and *WNT4* as risk factors for uterine fibroids and endometriosis and similarly report variants in loci on chromosome 6 encoding for *SYNE1* and *ESR1* as risk factors for both conditions. Interestingly, a third locus, on chromosome 2, is described as having opposing effects in both studies (*GREB1*). A fourth risk variant in on chromosome 11, *FSHB* was only identified in one of the two meta-analyses. While it is not yet clear how the variants in these genes might lead to an increased risk of developing endometriosis and/or uterine fibroids, a mechanistic connexion can be postulated more obviously in some cases than others: *CDC42* (cell division cycle 42) encodes a small Rho GTPase of a family of proteins regulating the cell cycle (39), while *WNT4* (Wingless-Type MMTV Integration Site Family, Member 4) encodes a ligand that interacts with the β-Catenin signalling pathway to control self-renewal in adult tissues by acting on somatic stem cells (40). Indeed, WNT4 has recently been shown to increase stem cell proliferation in uterine fibroids (41).

The genes on the other locus of significance identified in both studies are *SYNE1* (Synaptic Nuclear Envelope Protein 1) and *ESR1* (oestrogen receptor 1). The oestrogen receptor mediates the physiological function of oestrogen, and, interestingly, variants in this gene are associated with cancers of the endometrioid type and result in the expression of SYNE1 (42), a gene in turn associated with menstrual migraine (43). Variants in *GREB1* (Growth Regulating Oestrogen Receptor Binding 1) the product of which functionally interacts with the oestrogen receptor, have been described as associated with endometriosis risk before (44); however, the recent studies were inconclusive as to the contribution toward the risk for developing uterine fibroids. *FSHB* (Follicle Stimulating Hormone Subunit Beta) encodes for the glycoprotein secreted by the anterior pituary gland that in women regulates granulosa cells and follicular growth (45). Genotypic variants in *FSHB* together with variants in its receptor have been shown to influence the level of available FSH in serum (46), which could explain the contribution toward the risk of developing endometriosis and/or uterine fibroids.

## Cancer Risk

Described as benign tumours, uterine fibroids resemble the growth of malignant solid tumours in their dependence on driver mutations, angiogenic potential and clonal spread (47). Endometriosis on the other hand appears more akin to metastasis formation in that the peritoneal cavity can contain numerous endometriotic lesions (48). These in turn present in different shapes, forms, sizes and composition but it is unclear whether lesions necessarily progress from one form to another (49). However, with genetic variants in *ESR1* and *FSHB* linked to cancer, the most concerning comorbidity is the increased risk for ovarian, endometrial and breast cancer (30). Not all studies investigated the risk of these cancers, however, those that did (Table 3) found that endometriosis increased the relative risk of breast cancer ~1.4-fold, while a more pronounced effect was seen on the risk of ovarian cancer with an ~4-fold increase in relative risk, and endometrial cancer, 2.4-fold. The risk increased again with laparoscopically confirmed endometriosis. However, the absolute incidence of ovarian cancer remains very low, estimated at 1.31% in the general population and at 1.42% in endometriosis patients (50). Interestingly, a diagnosis of adenomyosis, where it was included in the analysis, decreased the risk of ovarian cancer (0.2-fold risk), while uterine fibroids increased the risk of breast cancer (OR 1.54 (95% CI 1.19–1.99) and ovarian cancer (OR 1.60 (95% CI 0.93–2.76) but decreased the risk of endometrial cancer (OR 0.78 (95% CI 0.35–1.74)). The most recent study (31) did not investigate comorbidity of uterine fibroids with cancers but shows that the presence of breast cancer in the baseline assessment of the study population increased the risk of developing uterine fibroids by 1.5-fold while the presence of cervical or ovarian cancer decreased that risk 5.5-fold and 2-fold, respectively.

**Table 3:** Comorbidity of endometriosis or uterine fibroids with breast, ovarian and endometrial cancers.

**References**	**Comorbidity**	**Breast cancer**	**Ovarian cancer**	**Endometrial cancer**
Nagai et al., (28)	Endometriosis	1.34 (0.91–1.96)	3.65 (2.16–6.19)	2.40 (1.14–5.04)
Surrey et al., (30)	Endometriosis	aHR 1.4 (1.1–1.7)	aHR 4.0 (2.8–5.7)	aHR 2.4 (1.6–3.8)
Surrey et al., (30)	Endometriosis (laparoscopically confirmed)	aHR 1.7 (1.1–2.8)	aHR 6.0 (2.4–15.5)	aHR 4.2 (1.4–12.2)
Choi et al., (29)	Endometriosis	No data	No data	No data
Choi et al., (29)	Adenomyosis	No data	0.2; *P* = 1.07 × 10^−6^	No data
Nagai et al., (28)	Uterine Fibroids	1.54 (1.19–1.99)	1.60 (0.93–2.76)	0.78 (0.35–1.74)

*aHR, adjusted hazard ratio (95% CI); all others, OR (95% CI)*.

The risk of uterine fibroids progressing toward leiomyosarcomata for their part is very low, with an incidence of 0.4 per 100,000 (51), vastly lower than the incidence of uterine fibroids. A study into the MED12 status of leimyosarcomata found MED12 exon 2 mutations in only 7% of tumours (52), which argues against this mutation as the driving force behind the malignancies. The minority developing into leiomyosarcomata could consist of a subtype of uterine fibroids marked by cells with aberrant nuclei which show declining MED12 expression at both mRNA and protein levels (53). This observation has led to MED12 being described as a tumour suppressor, with the mutation leading to uterine fibroid growth and complete loss giving rise to malignancy (54). The loss of MED12 would in any case not be sufficient unless accompanied by an additional genetic hit or instability as recently described in histone-mediated chromatin accessibility (37). Uterine fibroids are thus in principle able to undergo malignant transformation. Fortunately, this occurs extremely rarely, and the prediction of which uterine fibroids might become malignant over time remains challenging.

## Treatments and Risks

Endometriosis related pain and increased menstrual flow are treated with hormonal drugs. The same products treat UF-related heavy menstrual bleeding. Combined contraceptives (oestrogen and progesterone, oral, transdermal, vaginal ring) suppress cell proliferation and enhance apoptosis on eutopic endometrium (55). Progesterone-only products (oral, subcutaneous implant, intrauterine device) have anti-inflammatory features and they cause atrophy on both eutopic and ectopic endometrium (56). GnRH agonists suppress the function of hypothalamic-pituitary-ovarian axis and so forth cause a hypo-oestrogenic state, which in turn inactivates endometrium activity (11). Aromatase inhibitors suppress oestrogen synthesis in adipose tissue. Two aromatase inhibitors, anastrozole and letrozole, are effective in reducing endometriosis related pain and improving quality of life (57). Its use for treatment of UFs is only supported with insufficient evidence (58). Selective progesterone receptor modulators (SPRMs, mifepristone, ulipristal acetate and asoprisnil) are effective in treatment of UF. They reduce menstrual bleeding and improve quality of life (59). Considering the aberrant progesterone signalling, a shared common pathway in UFs and endometriosis, the effect of ulipristal acetate has been under investigation for endometriosis. To date, only limited evidence support its effectiveness in treating endometriosis related pain symptoms. Lesion size and weight were significantly decreased on a study on mouse model (60). Evidence on humans relies on a case report on a 3-month trial: A daily dose of 15 mg ulipristal improved pain symptoms (61).

Dysmenorrhea is treated with NSAIDs (non-steroidal anti-inflammatory drugs) (11, 62). Their mechanism of action is based on inhibition of cyclo-oxygenase enzymes, which in turn blocks the production of prostaglandins (63). Additionally, NSAIDs reduce menstrual bleeding (64). Endometriosis-related pain may also be treated with paracetamol, or selected cases with weak opioids, and neuropathic pain with anti-epileptics and anti-depressants. However, the pain and pressure symptoms caused by a fibroid-enlarged uterus cannot be permanently relieved medically (65).

Thus, surgery is a treatment option for both endometriosis and UF. The aim for endometriosis surgery is to relieve pain and improve fertility by removing all visible lesions or signs of disease (48), UF related symptoms such as heavy menstrual bleeding, pain, pressure and reduced fertility, can also be improved by surgery (5). The alternatives are resection of submucosal UF via hysteroscopy, myomectomy and hysterectomy, via either laparoscopy or abdominal laparotomy. The preoperative diagnosis for UFs is fairly accurate, as most patients have either TV-USS or MRI, which both have a high sensitivity for detecting UFs (66). The case for preoperative diagnosis of endometriosis is quite different, as only endometriomas and large adenomyotic nodules are detected on imaging without prior suspicion. Deep endometriosis lesions, such as rectovaginal, bowel, ureteric and uterosacral, require clinical experience in interpreting the functional TV-USS or MRI images, whereas small and peritoneal lesions are detectable only during surgery. Clinical suspicion is crucial in reducing interoperative incidental endometriosis diagnosis, in careful planning of the extensity of surgery, and in proper patient consenting.

## Conclusions

Endometriosis and Uterine Fibroids are the two most common gynaecological diseases affecting women's quality of life and uterine function. Data arising from recent studies show an association between them in shared, partly oestrogen-related pathophysiology resulting in an increased comorbidity risk of women with endometriosis to also have UF and vice versa. This has important implications for treatment of either condition. In the light of substantial comorbidity between uterine fibroids and endometriosis, it would be beneficial to include the option of concomitant surgery for endometriosis during resections of uterine fibroids. Treatment of one condition while ignoring the other could fail to address the patient's complaint. Especially in fertility patients, controlled ovarian stimulation could worsen an undetected endometriosis.

We hope that further research into the comorbidity and underlying commonalities of endometriosis and uterine fibroids will yield the means to causally address both conditions in the future, to increase and preserve women's health.

## Author Contributions

OU wrote the review and discussed the manuscript. HN wrote the manuscript. TT conceived of, wrote the review, and discussed the manuscript. All authors contributed to the article and approved the submitted version.

## Conflict of Interest

The authors declare that the research was conducted in the absence of any commercial or financial relationships that could be construed as a potential conflict of interest.

## Publisher's Note

All claims expressed in this article are solely those of the authors and do not necessarily represent those of their affiliated organizations, or those of the publisher, the editors and the reviewers. Any product that may be evaluated in this article, or claim that may be made by its manufacturer, is not guaranteed or endorsed by the publisher.
